# Immunological Changes in Mesothelioma Patients and Their Experimental Detection

**DOI:** 10.4137/ccrpm.s577

**Published:** 2008-03-26

**Authors:** Megumi Maeda, Yoshie Miura, Yasumitsu Nishimura, Shuko Murakami, Hiroaki Hayashi, Naoko Kumagai, Tamayo Hatayama, Minako Katoh, Naomi Miyahara, Shoko Yamamoto, Kazuya Fukuoka, Takumi Kishimoto, Takashi Nakano, Takemi Otsuki

**Affiliations:** 1 Department of Hygiene, Kawasaki Medical School, 577 Matsushima, Kurashiki 7010192, Japan; 2 Department of Respiratory Medicine, Hyogo Medical College of Medicine, 1-1 Mukogawa-cho, Nishinomiya, 6638131, Japan; 3 Okayama Rosai Hospital, 1-10-25 Chikkou-midori-machi, Okayama 7028055, Japan

**Keywords:** asbestos, immunology, mesothelioma, chrysotile

## Abstract

It is common knowledge that asbestos exposure causes asbestos-related diseases such as asbestosis, lung cancer and malignant mesothelioma (MM) not only in people who have handled asbestos in the work environment, but also in residents living near factories that handle asbestos. These facts have been an enormous medical and social problem in Japan since the summer of 2005. We focused on the immunological effects of asbestos and silica on the human immune system. In this brief review, we present immunological changes in patients with MM and outline their experimental detection. For example, there is over-expression of *bcl-2* in CD4+ peripheral T-cells, high plasma concentrations of interleukin (IL)-10 and transforming growth factor (TGF)-ß, and multiple over-representation of T cell receptor (TcR)-Vß in peripheral CD3+ T-cells found in MM patients. We also detail an experimental long-term exposure T-cell model. Analysis of the immunological effects of asbestos may help our understanding of the biological effects of asbestos.

## Introduction

It is common knowledge that asbestos exposure causes asbestos-related diseases such as asbestosis, lung cancer and malignant mesothelioma (MM) not only in people who have handled asbestos in the work environment, but also in residents living near factories that handle asbestos. These facts have been an enormous medical and social problem in Japan since the summer of 2005 (Kanazawa et al. 2006; Murayama et al. 2006; Nakano, 2006). Several patients with MM living in Amagasaki, Hyogo prefecture, Japan have featured in news reports. These patients resided 1 km from an asbestos factory and had no identifiable occupational exposure to asbestos. Given that MM is an incurable disease and prognosis is not promising (Vogelzang and Pass, 2006; Zucali and Giaccone, 2006; Tsiouris and Walesby, 2007), and considering the absence of effective government legislation concerning the usage of asbestos, people in Japan have become concerned about social and medical issues related to asbestos.

Asbestos is categorized as a silicate (mineralogical complexes containing metals, such as iron and magnesium) and includes forms such as chrysotile, crocidolite, and amosite. Patients exposed to asbestos develop pulmonary fibrosis known as asbestosis, mesothelial plaque and malignant diseases such as lung cancer and MM (Niklinski et al. 2004; Becklake et al. 2007; O’Reilly et al. 2007). The mechanisms of asbestos-induced carcinogenesis are thought to produce an accumulation of DNA damage due to asbestos-induced production of reactive oxygen/nitrogen species (ROS/RNS) and an escape from the asbestos-induced activation of the mitochondrial apoptotic pathway (Shukla et al. 2003; Upadhyay and Kamp, 2003). In addition, we believe that some of these malignancies may be caused by a decline in tumor immunity owing to exposure of immunocompetent cells to asbestos.

Silica is known as one of the strongest environmental substances that cause autoimmunity dysfunction (Hess, 2002; Cooper and Parks, 2004). Silicosis patients often develop immunological complications such as rheumatic arthritis (known as Caplan syndrome (Caplan, 1953)), systemic sclerosis (SSc), and systemic lupus erythematoses (SLE). The effects of silica on autoimmunity have also been recognized following the discovery that patients who receive plastic surgery with implants containing silicone ([SiO_2_-O-]_n_) show frequent complications involving autoimmune disorders (Shons and Schubert, 1992; Hirmand et al. 1993). These findings clearly indicate that crystalline silica causes dysregulation and/or disturbance of the human immune system, particularly autoimmunity.

The overall evidence suggests that asbestos may influence human immunocompetent cells and that such alterations may affect the occurrence and progression of asbestos-related malignant diseases. Thus, we have focused on the immunological effects of asbestos. Among the many types of asbestos, chrysotile has mainly been used in our experiments. It is known that magnesium, the main compartment of chrysotile as silicate, usually dissociates from the chrysotile core (SiO_2_) in the human body after inhalation, and chrysotile is known to induce malignant transformation. However, its carcinogenic capacity is lower than that of other forms of iron-containing asbestos such as crocidolite and amosite (Harington, 1991).

In this article, we present immunological changes in MM patients with our experimental model. These changes may have resulted from the immunological effects of asbestos on human immunocompetent cells, and may offer some suggestions for the immunological prevention of the occurrence and progression of asbestos-induced malignant diseases.

### *bcl-2* expression of peripheral CD4+ T cells in MM patients

**Figure 1.**
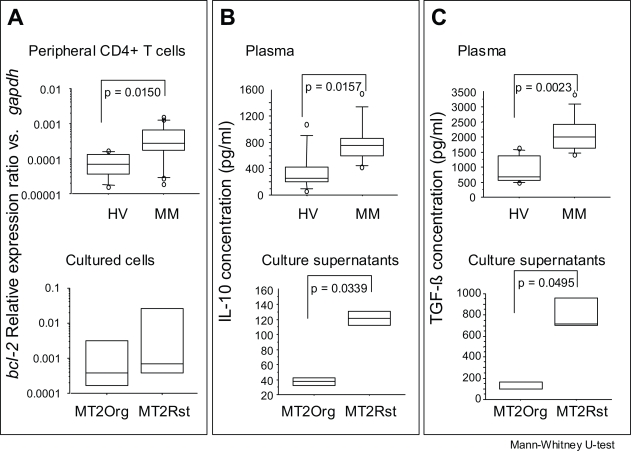
**Comparison of *bcl-2* relative expression ratio vs. *gapdh* plasma concentrations in anti-inflammatory cytokines in MM patients and healthy volunteers (HV), and *bcl-2* expression and secretion of these cytokines from experimental low-dose and long-term exposed T-cell models to asbestos (MT-2Org and MT-2Rst, see text for details).** Panel **A** shows the relative expression ratio of *bcl-2* in peripheral blood CD4+ cells (upper panel) from MM patients and HV, or in cultured MT-2Org and MT-2Rst cells (lower panel). Panels B and C show the plasma concentrations of IL-10 (**B**) and TGF-ß (**C**) from MM patients and HV (upper panels), or the concentrations in culture supernatants of IL-10 (**B**) and TGF-ß (**C**) from MT-2Org and MT-2Rst cells (lower panels). Peripheral blood mononuclear cells (PBMCs) were isolated from the heparinized blood of healthy donors and MM patients using a Ficoll-Hypaque density gradient (Separate-L^®^, Muto Pure Chemicals Co. Ltd., Tokyo, Japan). For the isolation of CD4+ T cells, PBMCs were further separated using Magnetic Cell Separation (MACS) CD4 MicroBeads (Miltenyi Biotech, Bergisch Gladbach, Germany) according to the manufacturer’s instructions. The enriched cells were >90% pure as determined by flow cytometry. Specimens were taken from healthy volunteers and patients from whom informed consent had been obtained. The Institutional Ethics Committee of Kawasaki Medical School, Hyogo College of Medicine, and Okayama Rosai Hospital approved the project. A fluorescence thermocycler (Mx3000P^®^ QPCR System, Stratagene Corporation, La Jolla, CA) was used for real-time RT-PCR experiments by following the instructions of the manufacturer. The fluorescence-labeled amplification product is measured continuously with this technique. Total RNA obtained from CD4+ T cells isolated from peripheral CD4+ T cells was extracted using an RNA Bee kit (Tel-Test, Inc., Friendswood, Texas), and 5 μg of RNA was reverse-transcribed with standard methods using a RevertAid™ H Minus First Strand cDNA Synthesis Kit (Fermentas International Inc., Ontario, Canada). An amount of cDNA equivalent to 50 ng of RNA served as the template for PCR in a volume of 20 μl (each primer and SYBER Premix Ex Taq, TaKaRa). The primers for *bcl-2* and *gapdh* were added to the same reaction tube at the optimal concentration for each primer set and PCR was performed. Primers were as follows: *bcl-2*; 5′-TGATGTGAGTCTGGGCTGAG-3′ (Forward: Fw) and 5′-GAACGCTTTGTCCAGAGGAG-3′ (Reverse: Rv), Bax; 5′-AGTAACATGGAGCTGCAGAGG-3′ (Fw) and 5′-ATGGTTCTGATCAGTTCCGG-3′ (Rv), *gapdh*; 5′-GAGTCAACGGATTTGGTCGT-3′ (Fw) and 5′-TTGATTTTGGAGGGATCTCG-3′ (Rv). The relative expression of various target genes such as *bcl-2* was calculated as follows when real-time RT-PCR was performed: [A: number of PCR cycles required to reach a certain intensity of fluorescence for the *gapdh* product. B: number of PCR cycles required to reach the same fluorescent intensity for the target gene product (*bcl-2*) derived from the same sample.] The relative level of the target gene is expressed as 1/2[B-A], with gapdh expression being 1.0. PCR products were confirmed to be successfully amplified by standard agarose gel electrophoresis and staining with ethidium bromide. Comparisons of the results for relative gene expression and proliferation assayed by real-time RT-PCR were analyzed using the Mann-Whitney U-test. Cytokines in plasma from MM patients and HV and culture supernatant were measured using an ELISA kit (Quantikine^®^ Human TGF-ß1 (or IL-10) Immunoassay; R&D Systems) and the Cytometric Bead Array of Human Th1/Th2 cytokine kit II (CBA, BD Bioscience, San Jose, CA, U.S.A.), and measurements were made using FACSCalibur flow-cytometry (BD Bioscience) according to the manufacturer’s instructions.

As shown in the upper panel of Figure 1-A, peripheral CD4+ T cells from MM patients showed a significantly higher expression of *bcl-2* compared to that of healthy volunteers (Miura et al. 2006). This may suggest that the over-expression of *bcl-2* in peripheral CD4+ T cells is one of the markers for the occurrence of MM, although it should be determined whether many cancer-bearing patients respond in a similar manner. The experimental background of this finding is as follows.

Experiments that exposed a high dose of chrysotile to peripheral fresh T cells or T cell-derived cell lines for a short time revealed that a human T-cell leukemia virus type-1 (HTLV-1)-immortalized human polyclonal T-cell line, MT-2, underwent apoptosis with ROS production via activation of the mitochondrial apoptotic pathway with the phosphorylation of p38 mitogen-activated protein kinase (MAPK) and c-Jun N-terminal kinase (JNK) signaling molecules. In addition, we observed a shift of the Bax-dominant Bax/Bcl-2 balance, the release of cytochrome-c from mitochondria into the cytosol, and the activation of caspases 9 and 3 upon short-term, high-level exposure to chrysotile (Hyodoh et al. 2005). However, we thought that an *in vitro* experimental model of chronic exposure was necessary in order to analyze the immunobiological effects of silicates during long-term exposure and to transfer these experimental findings to clinical analyses.

Thus, we established a chrysotile-induced apoptosis-resistant subline of MT-2 (MT-2Rst), and characterized the cell biological differences between the original MT-2 cell line (MT-2Org) and MT-2Rst. MT-2Rst cells were characterized by (i) an enhanced expression of *bcl-2* as shown in the lower panel of Figure 1-A, restoring apoptosis sensitivity with the decrease in *bcl-2* expression level by siRNA, (ii) excessive interleukin (IL)-10 secretion and expression, and (iii) the activation of signal transducers and activators of transcription (STAT) 3 inhibited by 4-amino-5-(4-chlorophenyl)-7-(t-butyl) pyrazolol [3,4-d] pyrimidine (PP2), a specific inhibitor of Src family kinases. These findings suggest that contact between cells and asbestos may affect the human immune system and trigger a cascade of biological events, such as the activation of Src family kinases, enhancement of IL-10 expression, STAT3 activation, and Bcl-2 over-expression as previously reported (Miura et al. 2006).

Another interesting finding was obtained from analyses using *bcl-2* expression in peripheral CD4+ T cells. We performed factor analysis using various clinical parameters and the *bcl-2* relative expression ratio (*bcl-2* RER) obtained by real-time RT-PCR from MM patients. Our results revealed that *bcl-2* RER, a past history of asbestos exposure, peripheral platelet counts, and serum CRP values formed one factor, and these parameters exhibited higher, present, lower count, and lower values, respectively, as shown in Table 1. Platelet-derived growth factor (PDGF) is one of the widely known MM-related growth factors and it functions as the autocrine/paracrine proliferation-promoting factor for mesothelioma cancer cells (Langerak et al. 1996, Klominek et al. 1998). Although higher serum levels of PDGF in MM patients are thought to be produced from mesothelioma cells and *bcl-2* RER is a marker of T cells chronically exposed to asbestos, these may be unknown biological mechanisms between immunocompetent cells with chronic exposure to asbestos and peripheral platelet counts via PDGF.

### IL-10 and TGF-ß concentrations in MM patients and the experimental model

As shown in the upper panels of Figures 1-B and 1-C, plasma concentrations of IL-10 and transforming growth factor (TGF)-ß were significantly higher in MM patients than in healthy volunteers. TGF-ß is known as one of the mesothelioma cell-producing cytokines (Gerwin et al. 1987; Maeda et al. 1994). However, the above-mentioned MT-2Rst cells, representing the outcome of the experimental low-dose and long-term asbestos-exposure T-cell model, showed significantly higher secretion of TGF-ß than MT-2Org cells (lower panel of Fig. 1-C). As we mentioned previously, the IL-10 concentration in culture supernatants of MT-2Rst was higher than that of MT-2Org (lower panel of Fig. 1-B). Thus, the source of the elevated TGF-ß and IL-10 concentrations in MM patients is not only tumor cells, but also immunocompetent cells.

It is interesting to note that IL-10 and TGF-ß are the important soluble factors necessary for the function of CD4+25+FoxP3+ regulatory T cells (Treg), even though cell-cell contact is the main route for the manifestation of Treg function (Wahl et al. 2004; Romagnasi, 2006). If circulating Treg and tumor-infiltrated Treg have enhanced function as a result of these elevated concentrations of IL-10 and TGF-ß, further aggressive progression of asbestos-induced cancer cells may have occurred. It may be important to analyze the Treg function using the experimental model that we have developed.

### T-cell receptor (TcR) Vß expression

**Figure 2.**
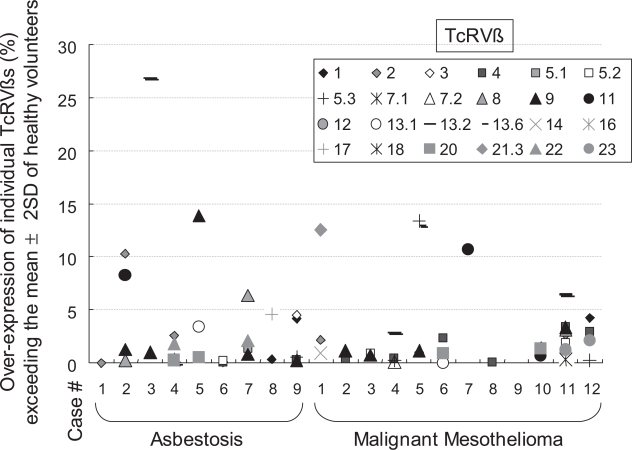
**TcRVß expression among patients with asbestosis and malignant mesothelioma.** Peripheral blood mononuclear cells (PBMCs) were obtained from 6 HV (mean age ± SD, 38.0 ± 6.4 years old; male(M):female(F),1:5), 9 asbestosis patients without significant clinical signs of complications such as lung cancer or malignant mesothelioma (ASB; 74.4 ± 3.9, all males), and 12 patients with malignant mesothelioma (MM; 58.7 ± 10.1, M:F, 9:3). Specimens were only taken once informed consent had been obtained. The study was approved by the Ethics Committee of Kawasaki Medical School, Okayama Rosai Hospital, Hyogo College of Medicine and Kusaka Hospital. The expression of TcRVß in CD3+ cells derived from HV, ASB and MM subjects was examined with an IOTest Beta Mark TcRVß repertoire analysis kit (Beckman Coulter, Inc., Fullerton, CA) using a FACSCalibur flow cytometer (Becton, Dickinson and Company, Franklin Lakes, NJ) according to the manufacturer’s instructions. This kit can analyze TcRVß 1, 2, 3, 4, 5.1, 5.2, 5.3, 7.1, 7.2, 8, 9, 11, 12, 13.1, 13.2, 13.6, 14, 16, 17, 18, 20, 21.3, 22 and 23 from 1 ml of peripheral blood. The 0% expression in this figure is the mean + 2SD for HV. Thus, each symbol indicates the number of over-expressions observed in individual patients for individual TcRVßs.

We reported previously that asbestos may act on peripheral T cells as a superantigen (Aikoh et al. 1998; Ueki, 2001). The effects of a superantigen such as staphylococcal enterotoxin B (SEB) may modify TcRVß on peripheral T cells to enhance multiple, but not clonal, TcRVß expression (Schubert, 2001; Li et al. 1999). As shown in Figure 2, various TcRVßs were over-expressed in MM and asbestosis patients. TcRVßs from most patients showed a higher expression, exceeding the average plus 2SD (standard deviation) limit. In addition, several TcRVßs such as Vß 1, 4 and 9 among the 24 kinds of TcRVß were strongly overexpressed in many patients. This phenomenon was also observed from the comparison of TcRVß expression in MT-2Org and MT-2Rst cell lines. As a result, MT-2Rst cells over-expressed various TcRVßs. Although TcRVß-over-expressing MT-2Org cells underwent apoptosis due to their first contact with chrysotile, MT-2Rst cells showed no significant changes when they again came in contact with chrysotile (Nishimura et al. 2006). These findings may suggest that the over-expression of various TcRVßs may be the result of contact between cells and chrysotile, an asbestos fiber, during the acquisition of resistance to CB-induced apoptosis caused by a long-term and low-dose exposure to CB (Nishimura et al. 2006).

The multiple over-expression of TcRVß in CD3+ peripheral T cells derived from asbestos-exposed patients may be one of the candidates to detect previous asbestos exposure. Although these data were obtained from a limited number of patients, it is worth continuing these analyses using samples from many patients in an effort to explore the biological mechanisms involved in these findings.

## Conclusion

**Figure 3.**
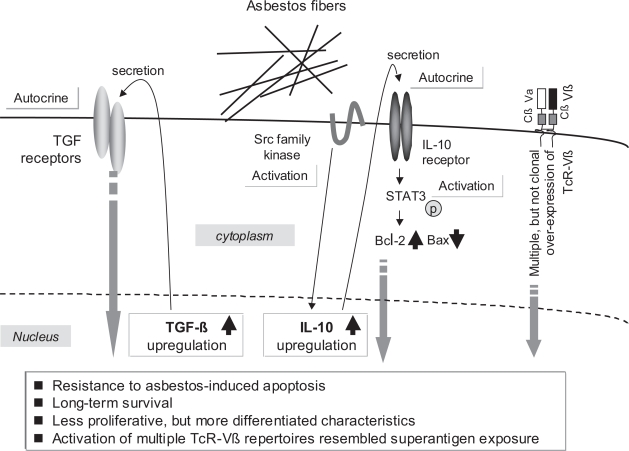
Experimental findings of immunological effects of chrysotile, a form of asbestos, induced by long-term and low-dose exposure using MT-2, an HTLV-1 immortalized human polyclonal T-cell line.

A summarized schematic presentation of various aspects of this investigation is shown in Figure 3. This schema only shows the experimental and clinical findings related to asbestos exposure of T cells. We have been investigating the effects of asbestos on the function of natural killer (NK) cells from cellular and molecular viewpoints. TGF-ß is similar to PDGF in that it is also known as a mesothelioma-producing growth factor (Gerwin et al. 1987; Maeda et al. 1994; Langerak et al. 1996; Klominek et al. 1998). Thus, the effects of TGF-ß1 on asbestos-exposed MT-2Rst cells are being investigated and compared with MT-2Rst and MT-2Org cells that have not been exposed to TGF-ß1. These examinations are on-going and will be presented in the near future.

Recent advances in immunomolecular studies have led to detailed analyses of the immunological effects of asbestos. Asbestos affects immunocompetent cells and these effects may be associated with the pathophysiological development of complications in asbestos-exposed patients such as malignant tumors. In addition, immunological analyses may lead to the discovery of new clinical tools for the modification of pathophysiological aspects of diseases, such as the regulation of tumor immunity using cell-mediated therapies, various cytokines and molecule-targeting therapies. As the incidence of asbestos-related malignancies increases against the growing concern in Japan since the summer of 2005 for medical and social problems created by such malignancies, efforts should be focused on developing a cure for these diseases in order to eliminate the nationwide anxiety concerning these malignancies.

## Figures and Tables

**Table 1.** Factor analysis of clinical parameters in mesothelioma patients with relative *bcl-2* expression in peripheral CD4+ T cells.

**Parameter**	**Value (a value of more than ± 0.4 is thought to contribute to the formation of this factor)**
*bcl-2* relative expression ratio in peripheral CD4+ T cells	**0.59009**
Histology (numbered) epithelial type = 1 mixed type = 2 sarcomatous type = 3	−0.14234
Past asbestos exposure (numbered) existence = 1 unknown = 2 none = 3	**0.55496**
White Blood Cell count	0.22054
Platelet count	**−0.76064**
Concentration of serum creatinine	0.21269
Concentration of serum CRP	**−0.79789**
Contribution rate	**19.18%**
